# Role of Sensory Nerves in Pulmonary Fibrosis

**DOI:** 10.3390/ijms25063538

**Published:** 2024-03-21

**Authors:** Charles E. Norton

**Affiliations:** Department of Medical Pharmacology and Physiology, University of Missouri, Columbia, MO 65212, USA; nortonce@missouri.edu

**Keywords:** pulmonary fibrosis, pulmonary hypertension, sensory nerves, cough, pericytes, edema sleep apnea, calcitonin gene-related peptide, substance P

## Abstract

Pulmonary fibrosis results from the deposition and proliferation of extracellular matrix components in the lungs. Despite being an airway disorder, pulmonary fibrosis also has notable effects on the pulmonary vasculature, with the development and severity of pulmonary hypertension tied closely to patient mortality. Furthermore, the anatomical proximity of blood vessels, the alveolar epithelium, lymphatic tissue, and airway spaces highlights the need to identify shared pathogenic mechanisms and pleiotropic signaling across various cell types. Sensory nerves and their transmitters have a variety of effects on the various cell types within the lungs; however, their effects on many cell types and functions during pulmonary fibrosis have not yet been investigated. This review highlights the importance of gaining a new understanding of sensory nerve function in the context of pulmonary fibrosis as a potential tool to limit airway and vascular dysfunction.

## 1. Introduction

Pulmonary fibrosis (PF) is a disease in which the lungs become scarred over time. It can result from occupational exposure, genetic defects, acute lung injury, or idiopathic causes. PF may also result from a secondary effect of other diseases, including autoimmune disorders and infections. This debilitating condition is associated with dyspnea, cough, and fatigue [1,2] resulting from impaired gas exchange caused by the excessive deposition of extracellular matrix components [3]. This is characterized by fibroproliferation and mononuclear inflammation. The incidence of PF has increased over the last several decades [4], which may be related to increased smoke and particle inhalation, as well as mineral and dust exposure associated with modern, urban lifestyles. The average life expectancy for an individual after being diagnosed with PF is 3 to 5 years [5]. There is currently no cure for the disease and limited therapy options. Thus, identifying treatments that prevent or slow the progression of this disease is vital to improving human health.

Sensory nerves are responsible for detecting harmful airborne stimuli and provide input to a variety of cells within the lungs, including airways and blood vessels. They play a critical role in regulating cardiopulmonary functions and maintaining homeostasis in healthy lungs. Alterations in the phenotype and sensitivity of these fibers are a hallmark of lung diseases, including asthma, viral infections, chronic obstructive pulmonary disease, and pulmonary fibrosis [6,7]. Despite such changes in function, sensory nerve signaling can downregulate PF [8,9], but the mechanisms through which sensory nerves are modified by and contribute to this disease are just beginning to be explored. Herein, we describe the mechanisms of sensory nerve function in the lungs and summarize current knowledge relating to the role sensory nerves play in airway and vascular smooth muscle cell (SMC) and endothelial cell (EC) function and consider questions that remain to be investigated. Furthermore, we explore how peptidergic nerve signaling may interact with supporting cells in the lungs, including fibroblasts, macrophages, pericytes, and lymphatic cells, which contribute to the pathology of PF.

## 2. Sensory Nerves in the Lungs

Within the lungs, innervation is most dense in extrapulmonary and hilar arteries [10,11,12]. The penetration of sympathetic and parasympathetic fibers varies between species but frequently stops shortly after the lung hilus [13,14]. Sensory/peptidergic fibers, in contrast, are found sparingly in vessels throughout the pulmonary vascular tree, airways, and alveoli [15,16,17,18,19]. Sensory nerves confer information about the local environment to the central nervous system, with their cell bodies located within the dorsal root ganglia [20]. Within the lungs, sensory (peptidergic) fibers play a vital role in regulating cardiopulmonary function under both healthy and disease conditions. These nerves are not homogenous in nature, having different anatomical and physiological phenotypes reflecting their location and purpose, and sensory nerve pattern and density vary with age, tissue, and vascular bed [21,22]. Each of these subtypes provides input to the central nervous system and is capable of driving cardiorespiratory reflexes. Unlike sympathetic nerves, sensory nerves can signal both antidromically and orthodromically, thus facilitating their participation in local axon reflexes independent of efferent signaling from the cell body [23]. Ergo, local stimuli experienced in the tissue, such as mechanical or chemical responses, can lead to neurotransmitter release and signaling independent of the central nervous system.

**Figure 1:**
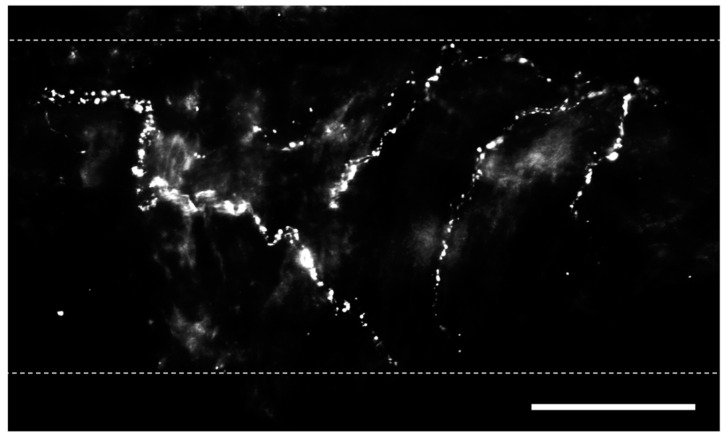
Perivascular sensory nerves on mouse pulmonary arteries. Representative calcitonin gene-related peptide (CGRP) staining (maximum z projections) on the surface of a ~100 µm pulmonary artery. Dotted lines indicate approximate vessel edge. Scale bar = 50 µm.

Bronchopulmonary sensory nerves are highly varied based on properties including location in the lungs, ganglionic origin, activation profile, conduction velocity, and responses that nerve activation elicits. Key classes of sensory fibers include nociceptors and mechanosensors. Stretch-sensitive mechanosensors are a group of afferent nerve fibers that respond to the nonharmful distension of the lungs that occurs during respiration [24]. The activation of these fibers is dependent on the rate and depth of breathing (i.e., tidal volume), and they can be grouped into rapidly adapting fibers located in the mucosal layer and slow-adapting fibers located in proximity to SMCs [25]. Nociceptors, in contrast, respond to lung injury and are classified into touch-sensitive cough fibers and bronchopulmonary C fibers [24]. Regardless of fiber type, pulmonary sensory nerves produce biologically active peptides, including substance P, neurokinin A, and calcitonin gene-related peptide (CGRP) [26], and immunostaining for these transmitters is utilized to identify sensory nerves (Figure 1) [19,27].

### 2.1. Calcitonin Gene-Related Peptide

CGRP is a 37-amino acid peptide that serves as the primary neurotransmitter released from sensory nerves [28] and is a potent vasodilator [29]. CGRP is synthesized in both central and peripheral sensory neurons and transported along axons in vesicles, where it is released [30]. Subsequently, CGRP can activate G-protein receptor-coupled CGRP receptors in airways and blood vessels [31,32]. These receptors are composed of calcitonin receptor-like receptor (CRLR), receptor activity-modifying protein 1 (RAMP1, the site of ligand binding and specificity), and receptor component protein [33]. CGRP signaling is terminated solely by degradation, as it does not undergo reuptake [28]. Although CGRP signaling can directly stimulate vasodilation in SMCs [31,34,35], an endothelium-dependent pathway that is mediated through nitric oxide signaling can also modulate this response [19,36].

Adenosine triphosphate (ATP) is released as a co-transmitter with CGRP [37,38]. ATP can activate two types of purinergic receptors, P2X and P2Y, which are located in airway and vascular cells [39,40]. Given the variety of receptor subtypes to which ATP can bind, purinergic signaling can lead to both contractile and dilatory effects on the target tissue [41]. Purinergic receptor expression also varies with vessel size [21]. Unlike CGRP, ATP, which is rapidly broken down into adenosine, can be reuptaken by the cell [42]. However, given that ATP can be released from sympathetic neurons erythrocytes, and other non-neuronal cell types in addition to sensory nerves, [43,44], it can be challenging to identify the source of ATP responsible for paracrine signaling.

### 2.2. Substance P

Substance P is synthesized in cell bodies within the dorsal root ganglia and transported in vesicles, along with CGRP and ATP, via axons to sensory nerve terminals [28,45]. Following its release, substance P elicits its effect by binding to neurokinin-1 receptors (NK1Rs) coupled to G-protein signaling in airways [46] and in vascular ECs [28,47]. Substance P does not undergo reuptake and, therefore, continues exerting its effects until undergoing enzymatic degradation [45]. Its vascular effects include hyperpolarization and increases in [Ca^2+^]_i_ in ECs, which activate endothelial nitric oxide synthase (eNOS) [47,48]. This causes hyperpolarization and vasorelaxation in SMCs. The dependency of this response on endothelial nitric oxide signaling is demonstrated by the loss of SMC hyperpolarization when the endothelium is disrupted or when eNOS is inhibited in isolated mesenteric arteries [47].

The physiological role of substance P in the vasculature remains controversial. Given that substance P has minimal effects on adjacent SMCs, the levels that reach endothelial cells are not sufficient to alter vessel diameter and permeability in all vascular beds [49]. The exogenous application of substance P has minimal effect on vessel diameter in mesenteric and hepatic vessels [50,51]; however, it can regulate the diameter of pulmonary arteries [52].

### 2.3. Neurokinin A

Neurokinin A is a 10-amino acid peptide that belongs to the same family of tachykinins as substance P. Neurokinin A has the highest affinity for neurokinin-2 receptors (NK2Rs) [53], although it is also capable of NK1R activation [54]. NK2Rs are present in airways [55], vascular SMCs [56], and ECs [57]. Neurokinin A is a potent bronchoconstrictor [58] but produces a modest pressor response in the vasculature [59]. Neurokinin A is proinflammatory and can also activate macrophages [54].

### 2.4. Interaction with Sympathetic Nerves

In addition to their direct effects on signaling, sensory perivascular nerves can interact through negative feedback to regulate sympathetic neurotransmission. The peptidergic transmitters CGRP and substance P can reduce the amplitude of sympathetically evoked vasoconstrictions [21]. These effects are mediated by the prejunctional inhibition of sympathetic nerve terminals without affecting downstream signaling pathways [60]. In a reciprocal manner, sympathetic perivascular nerves can inhibit the activity of sensory nerves [61]. In rat mesenteric arteries, norepinephrine (NE) acts on prejunctional α2 adrenoreceptors of sensory nerve terminals to reduce the release of CGRP [62]. ATP released by sympathetic nerves can also bind to P2Y receptors on peptidergic fibers to prevent CGRP signaling [63]. As the presence of sympathetic nerves in the lungs is relatively modest in many organisms, such effects likely vary across species.

## 3. Sensory Nerves in Airway and Cough

**Figure 2:**
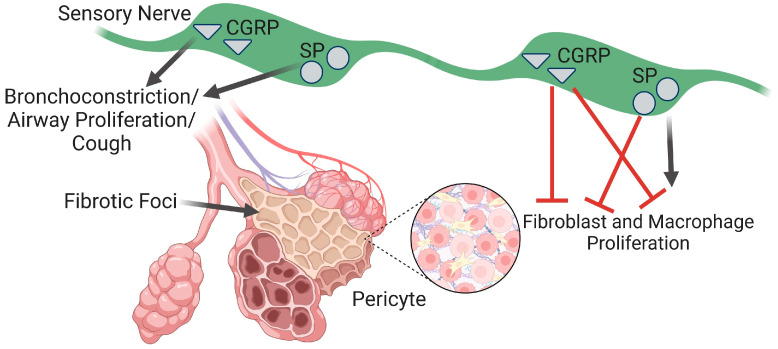
Summary of sensory nerve signaling in airways during pulmonary fibrosis (PF). Calcitonin gene-related peptide (CGRP) and substance P (SP) promote bronchoconstriction, airway proliferation, and chronic cough. Whereas CGRP limits the formation of fibrotic foci by attenuating fibroblast and macrophage activation, SP diminishes fibroblast signaling and potentiates macrophage inflammation. Image created with Biorender.

Excessive extracellular matrix accumulation in the lungs leads to the destruction of the alveolar structure, respiratory failure, and PF. Chronic inflammation and repeated injury to alveoli are vital to disease progression. Findings by Leslie et al. indicate that recurrent stretch injuries resulting from pressure changes during breathing can promote alveolar collapse, lung injury, and fibrotic cascade in PF [64]. Lung fibrosis has a heterogenous pattern with fibrotic lesions where collagen, fibroblasts, and myofibroblasts accumulate in the pulmonary interstitium [65]. Furthermore, the loss of gas exchange may be augmented by the loss of terminal bronchioles occurring within regions of minimal fibrosis [66]. Sensory nerves can influence various aspects of airway structure and function in the lung (Figure 2).

### 3.1. Sensory Nerves in Airway Structure and Function during PF

Sensory nerves are patterned after the airways in the lung with projections into the alveolar regions [14]. They can induce changes in respiratory behavior [67] and are important for airway responses to disease [68]. These nerves are capable of providing both acute signaling to the local respiratory tissue in addition to modulating responses via the central nervous system. Vagal sensory nerve perturbations are also critical in dyspnea [69]. CGRP promotes the growth of both bronchial [70] and alveolar [71] epithelial cells following lung injury. This growth may help mitigate the loss of airways during fibrosis and develop additional pathways for gas exchange in unaffected tissue. Substance P and neurokinin A promote smooth muscle migration and airway remodeling by enhancing tubulin expression via NK1R activation [72]. However, NK1R activation also has anti-proliferative effects on human airway smooth muscle cells [73].

Increased levels of sensory neuropeptides trigger inflammatory responses, mucus secretion, and bronchoconstriction [74], and the biogenesis of mucus is enhanced in PF [75]. CGRP itself is not an effective mediator of bronchoconstriction but can attenuate substance P-induced bronchoconstriction with a greater effect on distal (smaller) airways than proximal (larger) airways [76]. Substance P plays a key role in airway contraction [77] and mucus secretion [46]. Purinergic signaling plays a key role in pulmonary inflammation signaling through molecules such as interleukin 1α [42] and is important for mucociliary clearance [40]. The lung expression of neurokinin A and NK1Rs is elevated in a bleomycin model of guinea pig lung fibrosis [78]. Such effects would be expected to enhance bronchoconstriction, further impairing gas exchange resulting from PF. Central control of breathing changes during PF, altering ventilator mechanics via neural plasticity [7]. However, how such effects alter local signaling in the lung remains to be investigated.

Fibroblasts produce an interconnected network of extracellular protein fibers and connecting proteins that provide structure to tissue. This network is especially key in the lungs, which require the formation of stable alveoli that are capable of expanding and contracting during respiration yet are not so fragile that they collapse, preventing gas exchange. Transforming growth factor (TGF)-β promotes the differentiation of fibroblasts into myofibroblasts, contributing to subepithelial fibrosis and airway smooth muscle proliferation in asthma [79]. Myofibroblasts possess elevated extracellular matrix protein production; constitutively secrete and have enhanced sensitivity to cytokines, chemokines, and growth factors; and gain the expression of contractile proteins [80]. In idiopathic PF, enhanced IL-6 signaling stimulates TGF-β, leading to a transition into myofibroblasts [81]. Early events of fibroblast transition into the profibrotic state include the dysregulation of ribosomal and copper-binding proteins, leading to the formation of fibrotic foci [82]. Neuropeptides can directly modulate fibroblasts, and although CGRP can promote fibroblast proliferation and migration in wound healing [83], in bleomycin-induced PF, CGRP attenuates the fibroblast expression of smooth muscle α-actin, collagen I, and collagen II [9]. While effects on PF remain to be investigated, substance P downregulates collagen in human lung fibroblasts with a concomitant increase in matrix metalloproteinase-1 expression [84].

Macrophages are the most common immune cells in the lung and have been recognized as playing a major role in PF pathogenesis. Macrophages are the main source of TGF-β, a primary effector molecule leading to fibrosis, and promote fibroblast proliferation and collagen synthesis [65,85]. Macrophage-driven inflammation can be reduced by CGRP in rodent lungs [86,87]. In contrast, CGRP can also promote macrophage interleukin (IL)-6 expression [88]. Substance P can stimulate macrophages to enhance the release of tumor necrosis factor-α (TNF-α), IL-1, and IL-6 [89]. Neurokinin A activates nuclear factor (NF)-κB gene expression in macrophages promoting inflammation through extracellular signal-related kinases (ERKs) and phosphoinositide 3-kinase signaling [54]. This proinflammatory state can drive the expression of NK2Rs in cancer cells [90], and if such signaling occurs in the lung, it may result in a feed-forward response in order to potentiate lung inflammation. Consistently, an NKR antagonist can prevent oxidative stress and inflammation in macrophages [91]. Despite these critical roles for inflammation in PF, immunosuppressive and anti-inflammatory drugs have proven ineffective in treating PF [92].

### 3.2. Pulmonary Fibrosis and Cough

The primary function of cough is to protect the airway from noxious stimuli and the inhalation of irritants. The cough reflex is mediated by sensory nerves in the epithelium of the upper airway and diaphragm, which transduce signals through the vagus nerve to the brain [67,93]. This stimulates the cough response, whereby the efferent fibers of the vagus, phrenic, and spinal motor nerves stimulate the diaphragm and abdominal muscles, leading to cough [2]. Transient receptor potential channels present in sensory nerves serve as the main sensors implicated in the initiation of cough [94].

Excessive (chronic) cough is a symptom of chronic lung diseases due to the increased activation of sensory neural pathways [95]. Chronic cough is defined as a cough lasting for a minimum of eight weeks. As many as 80% of patients with idiopathic PF may experience chronic cough, and the presence of chronic cough serves as an independent predictor of disease progression [2,96]. Cough sensitivity to capsaicin is also enhanced in a guinea pig model of PF [78]. Key roles for CGRP, substance P, and neurokinin A contribute to this response. Furthermore, reflex cough sensitivity is increased in patients with PF [97]. This may be due to an upregulation of sensory nerve fibers within the lung. Sensory nerves are sensitive to mechanical changes and may be modified by the change in lung force induced by fibrosis [98,99]. Elevated levels of neurotrophins are also observed in PF [97,98]. Neurotrophins, a class of growth factors, can promote the development and survival of sensory nerves and enhance the cough reflex [2]. Such stimuli may also lead to changes in the central nervous system meant to modify behavioral cough [67,100]. Nerves that inhibit cough can also be reduced in PF, particularly where fibrosis is most severe [99]. Regardless of the origins of cough during PF, this effect enhances the patient burden and treatment requirements associated with this disease [101].

## 4. Sensory Nerves in Vascular Dysfunction

Interstitial lung diseases encompass a broad range of conditions that frequently lead to the development of PF [102]. Despite being a respiratory disease, increasing evidence indicates that there is an important vascular component to PF. While it was originally thought that PF-induced pulmonary vascular dysfunction solely resulted from the associated chronic hypoxia (CH), increasing evidence suggests that alternative mechanisms and signaling events are also involved [103,104]. The mortality of PF is predicted by the presence of pulmonary hypertension (PH), which develops over time in most patients with PF [105]. While most cases of PH with PF are mild, severe PH can also develop. Systolic pulmonary arterial pressure has a strong inverse correlation with survival. Specifically, patients with pulmonary arterial pressures exceeding 50 mmHg have a mean survival of less than one year compared with a survival of 4 years or more in patients with pressures lower than 50 mmHg [106]. Therefore, understanding how to delay the development of PH in PF has the potential to lead to therapies that extend survival and improve quality of life in afflicted patients.

**Figure 3:**
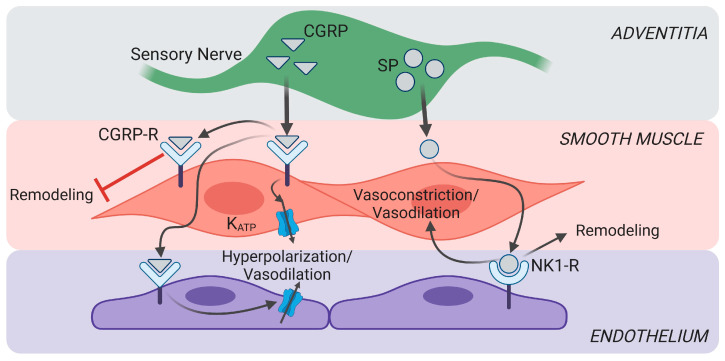
Summary of sensory nerve signaling in blood vessels during pulmonary fibrosis. Calcitonin gene-related peptide (CGRP) signaling promotes vasoconstriction and attenuates vascular remodeling in pulmonary hypertension (PH). In contrast, substance P (SP) can elicit vasoconstriction or dilation and can promote the proliferation of SMCs, leading to vascular remodeling. Whereas CGRP acts on both SMCs and ECs, SP acts solely on ECs. Image created with Biorender.

In addition to the varied origin and density of sensory fibers, differences in the size and location of nerve terminals in the vessel wall can impact vascular responses from sensory nerves. Due to greater adventitia, large arteries have greater diffusion distances (several hundred nanometers) than small arteries (~100 nm) [21,107], increasing the time to response and reducing the effectiveness of the released chemical concentration. These differences, combined with receptor expression and effector signaling pathways, tune vascular responses to respective neurotransmitters. At present, little attention has been paid to how PF alters the location, density, or diffusion distance of sensory nerve fibers. Given the ability of sensory nerve signaling to downregulate PF [8,9], this knowledge has the potential to significantly impact the treatment of this disease (Figure 3).

### 4.1. Pulmonary Fibrosis and Vascular Remodeling

Pulmonary vascular remodeling is characterized by the medial thickening of the vessel wall, which reduces arterial inner diameter and increases vascular resistance. This process can result from changes in the intima, media, and adventitia with a key contribution of inflammatory cells [108,109]. Given the enhanced role of inflammation in PF, as compared with other causes of PH, the role of inflammatory mediators in pulmonary vascular remodeling may be enhanced in this setting. Reduced lung volumes in PF may also raise pulmonary vascular resistance [110]. As functional residual capacity becomes impaired, extra-alveolar vessels become increasingly tortuous and may collapse. In a similar fashion, terminal airways can collapse in low lung volumes, which can potentiate alveolar hypoxia and hypoxic pulmonary vasoconstriction [111,112]. Anastomoses formed between bronchial/pleural arteries and pulmonary vasculature in fibrotic lungs [113] further complicate blood flow architecture in PF.

Rarefaction, or a reduction in the total number of blood vessels within the lung, can also increase pulmonary vascular resistance by reducing the number of parallel pathways for blood through the circulation of the lung. However, contradictory reports exist regarding whether fibrotic lungs are more [114,115] or less vascularized [116,117]. These discrepancies may be based on the model or species utilized or the region of the fibrotic lung that is studied. For instance, in human patients with idiopathic PF, capillarity is increased in nonfibrotic regions of the lung and greatly reduced in regions of fibrosis [118]. While capillaries are not typically sites of vascular resistance within the lung, these structural changes reduce perfusion to the affected regions of the lung [119]. Such a reduction in local perfusion may cause further changes in the local signaling milieu that contribute to disease. Conflicting reports exist as to the effectiveness of inhibiting the angiogenic remodeling of the pulmonary vasculature in PF. The early inhibition of angiogenesis with the chemokine CXCL11 reduced pulmonary collagen deposition in a preclinical mouse model of PF induced by bleomycin in [120]. In contrast, the depletion of vascular endothelial growth factor (VEGF)-A from inflammatory cells led to a reduction in pulmonary vasculature and reduced fibrosis in [121]. The differences in these findings may relate to the timing or pathway of inhibition. Nonetheless, vascular rarefaction has the potential to work synergistically with the medial remodeling of pulmonary arteries to increase pulmonary vascular pressure, thus contributing to PH associated with PF.

Vascular signaling may also regulate remodeling in the lungs. Studies by Oliveira and colleagues illustrate that chemokine receptor 2 (CXCR2) expressed on endothelial cells is reduced in lung tissues in patients with PH resulting from lung fibrosis [122]. In animal models, by using bleomycin to induce PF in wildtype and CXCR2 knockout mice, the authors demonstrated that reductions in CXCR2 signaling lead to the increased expression of matrix metalloproteinase 9 (MMP9), which has been previously implicated in lung remodeling in PF [123]. The full milieu of vascular signaling proteins that have the potential to contribute to vascular remodeling remains to be investigated, but it is likely that similar signaling effects can be observed.

In hypoxia-dependent PH, CGRP plays a therapeutic role, wherein prepro-CGRP reverses established pulmonary vascular remodeling, reduces pulmonary arterial pressure, and attenuates the development of PH [124,125]. Substance P levels have been reported to be elevated in animal models of PH, and elevated levels of substance P can elicit PH [126,127,128]. Furthermore, substance P leads to the proliferation of vascular smooth muscle cells, which leads to vascular remodeling [128]. While mechanisms of sensory nerve function in vessel architecture have been well established in hypoxia-dependent PH, their role in vascular remodeling during PF remains to be studied.

### 4.2. Changes in Vascular Tone in Pulmonary Hypertension and Pulmonary Fibrosis

Sensory perivascular nerves play an important role in maintaining low pulmonary vascular resistance. However, perivascular sensory nerve density is lower in pulmonary (Figure 1; ~6% vessel coverage) arteries compared with systemic (mesenteric (~25% vessel coverage) arteries [19,22]. A potential explanation for this reduced nerve density in pulmonary arteries may be that mesenteric arteries are also innervated by sympathetic nerves, which negatively inhibit sensory nerves through presynaptic inhibition via α_2_-adrenoreceptors [61]. As smaller pulmonary vessels typically lack sympathetic innervation [13], neurotransmitter release is not inhibited in this manner. Regional differences between the systemic and pulmonary circulation may also be attributed to the lungs receiving the entire cardiac output, whereas blood flow to respective beds of the systemic circulation is subject to redistribution to various organs.

We have characterized the mechanisms of CGRP-mediated vasorelaxation in the lungs [19]. For both SMCs and ECs, CGRP causes membrane hyperpolarization via the PKA-dependent activation of K_ATP_ channels, leading to a decrease in the vessel wall, [Ca^2+^]_i_, and pulmonary vasodilation (Figure 3). Furthermore, within the pulmonary circulation, there is a lack of contribution from K_Ca_ channels to this response, which is present in mesenteric arteries [19,47]. While the vasoactive effects of sensory nerves during pulmonary fibrosis remain to be characterized, the loss of CGRP dilation has been identified as a contributing factor in the development of CH-induced PH, yet whether this effect can be observed in PF, particularly prior to the onset of severe hypoxia, remains to be seen. The release of CGRP from the pulmonary vasculature is reduced in patients with PH induced by CH [129], and plasma CGRP levels are inversely correlated to systolic pressure in the main pulmonary artery [130]. Furthermore, vasodilator sensitivity to CGRP is reduced [131]. In conjunction with increased levels of sensory nerves promoting normal airway function during PF [97], vascular CGRP receptor levels are increased in hypoxia-induced PH [132], suggesting that this pathway is a key potential target for the treatment of PF.

In contrast to CGRP, substance P can act as a vasoconstricto in the pulmonary circulation. In addition, levels of substance P are elevated in experimental models of PH [133]. Other studies within dog lungs, however, indicate that substance P has minimal effects on vasomotor tone in normoxic lungs but can act as a dilator in the presence of hypoxia [52]. Reasons for the observed differences in these studies are not readily apparent but may relate to species, preparation, or disease context (i.e., chronic vs. short-term hypoxia). In patients with PH, substance P fails to elicit the endothelium-dependent dilation that can be observed in healthy subjects [131]. The effects of sensory nerves in the lung may be further complicated by signaling crosstalk between various neurotransmitters. In mesenteric arteries, substance P can diminish CGRP-mediated sensory vasodilation in inflammatory bowel disease [134], but signaling crosstalk in the lungs remains to be addressed. Further studies of sex differences in the context of PF-induced PH are also warranted given the protective role that estrogen plays in the development of PH [135], combined with sex-specific responses to sensory neurotransmitters [136].

## 5. Vascular Pericytes in Pulmonary Fibrosis

Pericytes are a population of mesenchymal cells located on the abluminal surface of blood vessels in a variety of tissues, including the lungs [137]. They play an essential role in vascular homeostasis and vessel remodeling [138]. Despite their location on the vessel exterior, they are capable of associating with ECs and influencing their function [139]. Pericytes have been implicated in numerous lung diseases including PF, PH, and asthma, due to their ability to differentiate into myofibroblasts that enhance collagen deposition and drive matrix remodeling [140]. Depending on the disease context, pericytes can lead to scar formation in either the subepithelium or the pulmonary arteries [141,142].

Despite clear evidence of the contribution of pericytes to pulmonary vascular disease, identifying their specific contributions remains an ongoing endeavor. Within the lung, pericytes share many common markers with fibroblasts such as CD73, CD90, CD44, PDGFRα, and CD105 in addition to sharing a similar morphology [138,140]. Pericytes from PH patients additionally have a high degree of transcriptional similarity to SMCs [143,144].

### 5.1. Pericytes in Airway Fibrosis

**Figure 4:**
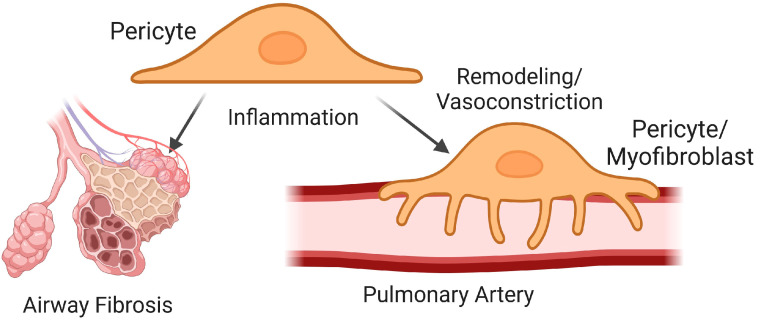
Summary of pericyte signaling during pulmonary fibrosis (PF). Inflammatory signaling leads to the transition of pericytes into myofibroblasts, which contribute to the formation of fibrotic foci and vascular remodeling in PF. Image created with Biorender.

Recent evidence has highlighted the contribution of pericytes to the formation of fibrotic tissue that occurs during PF (Figure 4). Studies on human lung pericytes show that these cells are particularly important for the formation of fibrotic foci localized between the lung epithelium and endothelium once they undergo a transition into a myofibroblast-like cell type [145], suggesting that matrix remodeling from these cells likely plays a key role in impaired gas exchange during PF. Despite their new characteristics, this population of myofibroblasts retains the ability to interact with the endothelium, demonstrating that these cells retain aspects of pericyte function as well [145]. Furthermore, these cells are highly resilient to eradication when targeting them for apoptosis. Therefore, identifying how to prevent their transition into myofibroblasts is vital to ameliorating their role in disease progression.

Inflammation is a common characteristic of pulmonary vascular disease [146] and is a key signaling event contributing to the role of pericytes in tissue fibrosis. This local tissue environment leads to pericyte uncoupling from vessels and facilitates their differentiation into myofibroblasts and the remodeling of the extracellular matrix [147,148]. This is driven by factors such as IL-6, IL-10, and TBF-β and chemokines such as CXCL12 in the lung [145,148,149,150]. Furthermore, inflammation induces the chemotactic migration of pericytes into inflamed areas of the lung [151], which will exacerbate the formation of pericyte-derived myofibroblasts. Following bleomycin lung injury, Foxd1 progenitor-derived pericytes expand and activate the expression of collagen and αSMC actin in fibrotic foci, leading to their transition into myofibroblasts [152].

### 5.2. Pericytes in Vascular Remodeling

Pericytes compose a significant portion of the remodeled hypertensive pulmonary arterial wall [143]. The transition into myofibroblast lineages also contributes to the excessive muscularization of large vessels in CH-induced PH [144]. The disruption of pericyte recruitment can attenuate pulmonary arterial muscularization, reduce disease severity, and restore hemodynamic parameters in the lung [153]. In addition, increased pericyte recruitment to vessels caused by a loss of prolyl hydroxylase domain protein 2 in ECs leads to perivascular fibrosis, right ventricular hypertrophy, and PH independent from any other stimuli [140]. Hypertrophic airway smooth muscles can limit apoptosis in neighboring myofibroblasts [154], and such effects may further the vascular remodeling observed in pulmonary vessels.

Pericytes are important regulators of angiogenesis [144,155] and may contribute to the differential response between the proangiogenic response in the nonfibrotic region of the lungs and vessel rarefaction in the fibrotic region of the lungs. Pericytes express VEGF receptors, which can stabilize vascular integrity and promote endothelial tube formation in the brain [140,156]. In contrast, findings from Yuan et al. illustrate that pericyte dysfunction following CH contributes to the loss of microvessels in the lung [144]. This is associated with a loss of Wnt5a in ECs, which diminishes healthy pericyte recruitment and the associated angiogenesis.

### 5.3. Pericytes in Vasomotor Control

Pericytes are also capable of regulating arterial diameter. They express the contractile proteins SM22 and α-smooth muscle actin [137,140]. Perivascular pericytes can increase contractile forces produced by SMCs [140]. Pericyte dysfunction in cancer has been linked to changes in metabolism whereby elevated hexokinase-2-mediated glycolysis upregulates Rho kinase-dependent contractility, leading to enhanced contractile activity in the lungs [157]. In human lungs, pulmonary hypertension increases pericyte coverage in the microvasculature, which normally has minimal smooth muscle investiture, and may promote greater contractility in these vessels [150].

Under healthy conditions, pericytes contain guanylyl cyclase signaling and are capable of responding to nitric oxide [158]. In a bleomycin model of PF in mice, pericyte-derived myofibroblasts that occupy former alveolar cavities lose guanylyl cyclase expression [159]. The loss of this signaling in these myofibroblasts can promote a procontractile state. Further investigation is required to delineate the exact mechanisms by which PF alters pericyte tone, but a combination of enhanced constrictor and impaired dilator functions may work synergistically to enhance pulmonary pericyte tone.

### 5.4. Sensory Nerve Modulation of Pericytes

Presently, the role of sensory nerves in regulating pericytes in the lungs is poorly understood. In the brain, the release of neurotransmitters can induce pericyte contraction or relaxation depending on the signaling molecule released [160]. It is also possible for pericytes to influence neuronal function through the release of neurotrophic factors such as glial cell line-derived neurotrophic factor [161]. Substance P can promote nonpathological vascular pericyte recruitment to blood vessels in mice [162] and disrupt the blood–brain barrier [163]. In the pancreas, exocrine pericytes respond to CGRP, but islet pericytes do not, highlighting the heterogeneity of various pericyte populations [164]. How peptidergic signaling during PF affects the various pericyte populations requires future study.

## 6. Pulmonary Fibrosis and Fluid Clearance

The lung must tightly regulate fluid clearance to prevent infection and maintain effective gas exchange. Conditions that lead to pulmonary inflammation, such as smoke exposure and PH, increase fluid extravasation from capillaries, which results in edema if it overwhelms the lymphatic capacity for clearance [165]. Pulmonary microvascular permeability can be increased in lung injuries, and CGRP contributes to the enhanced fluid flux [166]. Substance P also increases vascular permeability by eliciting changes in endothelial structure and function mediated by NO signaling [167,168]. The role of pericyte dysfunction during PF, as described above, can also contribute to pulmonary edema. Pericytes in the lung regulate capillary permeability, and defective pericytes increase leaky vessels [157,169]. Such changes in permeability are regulated by altered metabolism via increased glycolysis and changes in c-Met and angiopoietin signaling. Activation of sensory nerves also results in hyperpermeability in the bronchial circulation [170]. The pulmonary lymphatic system is responsible for preventing fluid accumulation and associated impairment of respiratory function.

### 6.1. Pulmonary Lymphatics

Normal lung parenchyma typically has a low investiture of lymphatic vessels. Most of these vessels lie in proximity to major blood vessels [171,172,173], although some have been observed in the alveolar space separated by a thin layer of connective tissue [174]. The lung provides a unique physiological challenge for lymphatic vessels, as the hydraulic pressure of the pleural fluid is subatmospheric. Thus, pleural lymphatics possess negative intraluminal pressure, which varies during the respiratory cycle [175]. Regardless of the unique challenges faced by lymphatic vessels in the lung, lymphatic dysfunction has identified roles in a variety of respiratory disorders affecting the lung [176] and is emerging as a key contributor to the pathogenesis of PF [177].

### 6.2. Lymphangiogenesis in PF

Interstitial PF leads to the development of newly formed lymphatic vessels in the alveolar space [178] and the fibrotic foci [179]. Lymphangiogenesis occurs early in the disease and in areas with relatively few abnormalities [178]. In this setting, fragmented hyaluronic acid associated with inflammation leads to the transdifferentiation of alveolar macrophages from human subjects with idiopathic PF as opposed to the more classical form of lymphangiogenesis, by which new vessels sprout from existing tissue [180]. Furthermore, the size of lymphatic vessels increases with disease severity [178]. Following lung injury, lymphatic proliferation can ameliorate PF [181]. In contrast, Ebina and colleagues observed a decrease in subpleural and interlobular lymphatics in idiopathic PF [179]. The reason for differences in these responses between investigators is unknown but may be related to disease severity or location, i.e., fibrotic or nonfibrotic tissue, in the lung. Although lymphangiogenesis has the potential to limit edema associated with PF and ameliorate lung injury, it may also be detrimental overall to lung function, as lymphangiogenesis in the lung is resistant to regression once the process has begun [182].

### 6.3. Lymphatic function in PF

In addition to structural changes in lymphatic vessels during PF, there may also be functional impairments. Impaired lymphatic function in mice leads to hypoxia [183]. Furthermore, lymphatic vessel dysfunction leads to many complications linked to PH [184]. In the lungs of patients with interstitial fibrosis, the lymphatic vasculature becomes fragmented [179], which may impair the ability of lymph to move out of the lung. VEGF signaling is required for the formation of button junctions in lymphatic capillaries, which permit the absorption of fluid and large macromolecules from the interstitium as a function of the relationship between intratympanic and interstitial pressure [185]. Given that levels of VEGF are reduced in PF [121], the impairment of macromolecule clearance (such as hyaluronans) observed in lung fibrosis may be due to a defect in lymphatic capillary function.

In mammals, lymph transport is typically mediated by extrinsic forces such as tissue compression or changes in interstitial pressure and the intrinsic contractile activity of lymphatic collecting vessels. The phasic contractions of collecting lymphatics allow for the propulsion of lymph against hydrostatic pressure gradients to the collection point in the subclavian veins [186,187]. Efficient lymph transport requires robust contractions across lymphatic muscle cells in addition to secondary lymphatic valves [188]. Deficits in lymphatic contractile activity contributing to lymphedema in the leg include irregular contractile rhythms and decreased contractile magnitude [189]. While little is known about lymphatic contractile activity in the lung, in other tissue beds, increases in intracellular Ca^2+^ are important in driving lymphatic pacemaker activity and contraction, and these processes are reliant on inositol triphosphate (IP3) receptors and the Ca^2+^-activated Cl^−^ channel TMEM16A [190,191]. TMEM16A is overexpressed in pulmonary arterial smooth muscle cells in patients with idiopathic PH and contributes to vasoconstrictor and vascular remodeling responses [192], with similar effects observed in rodent models of PH [193,194]. Lung lymphatic collecting vessels from mice have minimal smooth muscle cells to drive lymph propulsion, suggesting the forward fluid flow is primarily driven by changes in pressure that occur during respiration [183]. However, the cannulation of sheep pulmonary lymphatic ducts showed pressure spikes indicative of contractions in [195], and myosin-stained muscle cells were observed circumferentially around lymphatic collecting vessels in [196]. Thus, the mouse may be an outlier with regard to the minimal muscularization of lymphatic vessels.

The presence of intraluminal valves is vital to preventing retrograde lymph flow. Lymphatic valves are formed in response to oscillatory shear stress, which modifies gene transcription [197]. The dysfunction of lymphatic valves is a main form of lymphatic dysfunction. For instance, the back leak of pressure limits the effectiveness of lymphatic flow in popliteal lymphatics, contributing to obesity-induced lymphedema [198]. Impairments in valve formation and function remain to be investigated in PF but could have marked effects on lymphatic drainage from the lung. The increase in diameter associated with pulmonary lymphatic dysfunction is likely impairs valve function unless there is a commensurate change in leaflet length, as the effectiveness of valve gating caused by pressure is a function of diameter and leaflet length. Fibrosis in leaflet/pre-collecting vessels could also affect valve efficiency.

### 6.4. Contribution of Lymphatic Cells to PF

Lymphatic disruption may also contribute to the fibrotic process. Chemokines such as CCL21 that are released by lymphatic ECs are elevated in patients with PF [178,199]. In addition to the ability of CCL21 to form lymphatic bodies [200], this cytokine can additionally promote the proliferation of fibroblasts in PF [201]. The disruption of lymphatic function can also contribute to alveolar damage [183], although whether this contributes to the loss of alveoli in PF remains to be established. PF also leads to abnormal mural cell/fibroblast recruitment to lymphatic space, which contributes to impaired drainage [177] and may further promote the development of fibrosis in the lung. Further research is required to fully understand the crosstalk between lymphatic vasculature, fibroblasts, and other cells, contributing to obtaining a greater understanding of the role of the lymphatic circulation in the development of PF.

### 6.5. Sensory Nerves and Lymphatic Function

As lymphatic vessels are composed of ECs and SMCs, they have the potential to respond to sensory nerve stimulation in a similar manner to blood vessels. Although the sensory innervation of lymphatic vessels within the lung remains to be fully delineated, sensory nerves lie in close proximity to lymphatic capillaries [32]. In other tissues, CGRP and substance P released by peptidergic fibers within the skin may be transported within the draining lymph to affect lymphatic vessel function [202]. Sensory nerves can regulate transcriptional dynamics in lymphatic tissue and regulate immune function [203,204]. In systemic lymphedema, CGRP ameliorates lymphedema by enhancing lymphatic capillary formation and regulates macrophage recruitment [205]. Presently, there is a paucity of data on how peptidergic signaling alters lymphatic function in the lung and during PF; however, based on findings in other tissues, this is a key topic for future investigation.

## 7. Sleep Apnea and Pulmonary Fibrosis

Sleep apnea is a serious sleep disorder in which breathing repeatedly stops and restarts. Sleep apnea is characterized by its cause: central apnea when the brain does not send signals required for breathing and obstructive apnea when the airway becomes blocked, stopping airflow. Sleep apnea impairs sensory nerve function in human patients [206], which may alter how the previously described pathways respond during PF.

Obstructive sleep apnea has an increased prevalence in idiopathic PF [207,208] and leads to additional difficulties such as cognitive impairment [209]. Sleep apnea may promote fibrotic mechanisms [210]. Repetitive forced inspiration against the closed glottis decreases lung interstitial pressures, which can result in alveolar deformation and proinflammatory vascular responses [211,212]. Various effects of PF, such as chronic cough, are also enhanced in sleep apnea [213]. Furthermore, intermittent hypoxia associated with obstructive sleep apnea has been linked to the development of PH through distinct mechanisms that notably differ from CH [214,215]. Therefore, in patients with PF and sleep apnea, a combination of chronic and intermittent hypoxia mechanisms in PH may further exacerbate these diseases. Early diagnosis and treatment of sleep apnea has been linked to improved mortality in patients with idiopathic PF [210]. Whether this improvement in patient outcome is mediated through better nerve, airway, or vascular function remains to be determined.

## 8. Conclusions

Sensory nerves are important regulators of bronchial tone, cough, and pulmonary vascular remodeling and diameter. Therefore, the modulation of sensory nerve signaling pathways has the potential to treat PF in a multifaceted fashion. However, as this review summarizes, they are capable of playing both beneficial and detrimental roles in disease development. The varied effects of CGRP, substance P, and neurokinin A require careful consideration when attempting to evoke a single effect. For instance, while CGRP-dependent dilation may attenuate PH associated with PF and limit bronchoconstriction [76], it also contributes to airway hyperemia in the bronchial circulation and promotes fluid flux into the lungs [166]. Therefore, localizing treatment to specific regions of the lungs is vital for patient care. Although oral antifibrotic drugs can attenuate symptoms and improve quality of life, lung transplantation remains the only treatment for idiopathic PF that increases life expectancy [102,216]. Given the limited availability of organs for transplantation, new avenues for drug therapy are clearly needed. Further studies identifying specific molecular mechanisms of sensory nerves may assist in developing novel therapies for this debilitating disease.

## Data Availability

Not applicable.

## References

[1] Mann J., Goh N.S.L., Holland A.E., Khor Y.H. (2021). Cough in Idiopathic Pulmonary Fibrosis. Front. Rehabil. Sci..

[2] van Manen M.J.G., Birring S.S., Vancheri C., Cottin V., Renzoni E.A., Russel A., Wijsenbeek M.S. (2016). Cough in idiopathic pulmonary fibrosis. Eur. Respir. Rev..

[3] Behr J., Ryu J.H. (2008). Pulmonary hypertension in interstitial lung disease. Eur. Respir. J..

[4] Marshall D.C., Salciccioli J.D., Shea B.S., Akuthota P. (2018). Trends in mortality from idiopathic pulmonary fibrosis in the European Union: An observational study of the WHO mortality database from 2001–2013. Eur. Respir. J..

[5] Ryu J.H., Colby T.V., Hartman T.E. (1998). Idiopathic pulmonary fibrosis: Current concepts. Mayo Clin. Proc..

[6] Mazzone S.B., Undem B.J. (2016). Vagal afferent innervation of the airways in health and disease. Physiol. Rev..

[7] Yegen C.H., Marchant D., Bernaudin J.F., Planes C., Boncoeur E., Voituron N. (2023). Chronic pulmonary fibrosis alters the functioning of the respiratory neural network. Front. Physiol..

[8] Hartopo A.B., Emoto N., Vignon-Zellweger N., Suzuki Y., Yagi K., Nakayama K., Hirata K. (2013). Endothelin-converting enzyme-1 gene ablation attenuates pulmonary fibrosis via CGRP-cAMP/EPAC1 pathway. Am. J. Respir. Cell Mol. Biol..

[9] Li X.W., Li X.H., Du J., Li D., Li Y.J., Hu C.P. (2016). Calcitonin gene-related peptide down-regulates bleomycin-induced pulmonary fibrosis. Can. J. Physiol. Pharmacol..

[10] El-Bermani A.W., Bloomquist E.I., Montvilo J.A. (1982). Distribution of pulmonary cholinergic nerves in the rabbit. Thorax.

[11] Fisher A.W.F. (1965). The intrinsic innervation of the pulmonary vessels. Acta Anat..

[12] Haberberger R., Schemann R., Sann M., Kummer W. (1997). Innervation pattern of guinea pig pulmonary vasculature depends of vascular diameter. J. Appl. Physiol..

[13] Cech S. (1973). Cholinesterase-containing nerve fibres on blood vessels in lungs of some laboratory mammals. Z. Zellforsch. Mikrosk. Anat..

[14] Su Y., Barr J., Jaquish A., Xu J., Verheyden J.M., Sun X. (2021). Identification of lung innervating sensory neurons and their target specificity. Am. J. Physiol. Lung Cell. Mol. Physiol..

[15] Cadieux A., Springall D.R., Mulderry P.K., Rodrigo J., Ghatei M.A., Terenghi G., Bloom S.R., Polak J.M. (1986). Occurrence, distribution and ontogeny of CGRP immunoreactivity in the rat lower respiratory tract: Effect of capsaicin treatment and surgical denervations. Neuroscience.

[16] Komatsu T., Yamamoto M., Shimokata K., Nagura H. (1991). Distribution of substance P-immunoreactive and calcitonin gene-related peptide-immunoreactive nerve in normal human lungs. Int. Arch. Allergy Immunol..

[17] Kummer W.A., Fischer A., Kurkowski R., Heym C. (1992). The sensory and sympathetic innervation of guinea-pig lung and trachea as studied by retrograde neuronal tracing and double-labelling immunohistochemistry. Neuroscience.

[18] Martling C.R., Matran R., Alving K., Hökfelt, Lundberg J.M. (1990). Innervation of lower airways and neuropeptide effects on bronchial and vascular tone in the pig. Cell Tissue Res..

[19] Norton C.E., Segal S.S. (2018). Calcitonin gene-related peptide hyperpolarizes mouse pulmonary artery endothelial tubes through K_ATP_ activation. Am. J. Physiol. Lung Cell. Mol. Physiol..

[20] Hobara N., Nakamura A., Ohtsuka A., Narasaki M., Shibata K., Bomoita Y., Kawasaki H. (2004). Distribution of adrenomedullin-containing perivascular nerves in the rat mesenteric artery. Peptided.

[21] Westcott E.B., Segal S.S. (2013). Perivascular innervation: A multiplicity of roles in vasomotor control and myoendothelial signaling. Microcirculation.

[22] Boerman E.M., Segal S.S. (2016). Depressed perivascular sensory innervation of mouse mesenteric arteries with advanced age. J. Physiol..

[23] Yabrak M. (2008). The axon reflex. Neuroanatomy.

[24] Undem B.J., Kollarik M. (2005). The role of vagal afferent nerves in chronic obstructive pulmonary disease. Proc. Am. Thorac. Soc..

[25] Sant’Ambrogio G., Widdicombe J.G. (2001). Reflexes from airway rapidly adapting receptors. Respir. Physiol..

[26] Kummer W.A. (2011). Pulmonary vascular innervation and its role in responses to hypoxia: Size matters!. Proc. Am. Thorac. Soc..

[27] Grasby D.J., Morris J.L., Segal S.S. (1999). Heterogeneity of vascular innervation in hamster cheek pouch and retractor muscle. J. Vasc. Res..

[28] Brain S.D., Grant A.D. (2004). Vascular action of calcitonin gene-related peptide and adrenomedullin. Physiol. Rev..

[29] Brain S.D., Williams T.J., Tippins J.R., Morris H.R., MacIntyre I. (1985). Calcitonin gene-related peptide is a potent vasodilator. Nature.

[30] Kashihara Y., Sakaguchi M., Kuno M. (1989). Axonal transport and distribution of endogenous calcitonin gene-related peptide in rat peripheral nerve. J. Neurosci..

[31] Bell D., McDermott B.J. (1996). Calcitonin gene-related peptide in the cardiovascular system: Characterizations of receptor populations and their (patho)physiological signaificance. Am. J. Physiol. Lung Cell. Mol. Physiol..

[32] Dakhama A., Larsen G.L., Gelfand E.W. (2004). Calcitonin gene-related peptide: Role in airway homeostasis. Curr. Opin. Pharmacol..

[33] McLatchie L.M., Fraser N.J., Main M.J., Wise A., Brown J., Thompson N., Solari R., Lee M.G., Foord S.M. (1998). RAMPs regulate the transport and ligand specificity of the calcitonin-receptor-like receptor. Nature.

[34] Nelson M.T., Huang Y., Brayden J.E., Herscheler J., Standen N.B. (1990). Arterial dilations in response to calcitonin gene-related peptide involve activation of K^+^ channels. Nature.

[35] Wellman G.C., Quayle J.M., Standen N.B. (1998). ATP-sensitive K^+^ channel activation by calcitonin gene related peptide and protein kinase A in pig coronary arterial smooth muscle. J. Physiol..

[36] Wisskirchen F.M., Burt R.P., Marshall I. (1998). Pharmacological characterization of CGRP receptors mediating relaxation of the rat pulmonary artery and inhibition of twitch responses of the rat vas deferens. Br. J. Pharmacol..

[37] Kawasaki H., Takasaki S., Saito A., Goto K. (1988). Calcitonin gene-related peptide acts as a novel vasodilator neurotransmitter in mesenteric resistance vessels of the rat. Nature.

[38] Holton P. (1959). The liberation of adenosine triphosphate on antidromic stimulation of sensory nerves. J. Physiol..

[39] Burnstock G. (2007). Physiology and pathophysiology of purinergic neurotransmission. Physiol. Rev..

[40] Thompson R.J., Sayers I., Kuokkanen K., Hall I.P. (2021). Purinergic receptors in the airways: Potential therapeutic targets for asthma?. Front. Allergy.

[41] Burnstock G. (2009). Purinergic regulation of vascular tone and remodelling. Auton. Autacoid Pharmacol..

[42] Le T.T.T., Berg N.K., Harting M.T., Li X. (2019). Purinergic signaling in pulmonary inflammation. Front. Immunol..

[43] Kennedy C., Saville V.L., Burnstock G. (1986). The contributions of noradrenaline and ATP to the responses of the rabbit central ear artery to sympathetic nerve stimulation depend on the parameters of stimulation. Eur. J. Pharmacol..

[44] Lohman A.W., Billaud M., Isakson B.E. (2012). Mechanisms of ATP release and signalling in the blood vessel wall. Cardiovasc. Res..

[45] White J.D., Stewart K.D., Krause J.E., McKelvy J.F. (1985). Biochemistry of peptide-secreting neurons. Physiol. Rev..

[46] Sponchiado M., Liao Y.S., Atanasova K.R., Collins E.N., Schurmann V., Bravo L., Reznikov L.R. (2021). Overexpression of Substance P in pig airways increases MUC5AC through an NF-kβ pathway. Physiol. Rep..

[47] Norton C.E., Boerman E.M., Segal S.S. (2021). Differential hyperpolarization to substance P and calcitonin gene related peptide in smooth muscle versus endothelium of mouse mesenteric artery. Microcirculation.

[48] Brain S.D., Cox H.M. (2006). Neuropeptides and their receptors: Innovative science providing novel therapeutic targets. Br. J. Pharmacol..

[49] Brain S.D. (1997). Sensory neuropeptides: Their role in inflammation and wound healing. Immunopharmacology.

[50] Li Y.J., Duckles S.P. (1992). Effect of endothelium on the actions of sympathetic and sensory nerves in the perfused rat mesentery. Eur. J. Pharmacol..

[51] Phillips J.K., Hickey H., Hill C.E. (2000). Heterogeneity in mechanisms underlying vasodilatory responses in small arteries of the rat hepatic mesentery. Auton. Neurosci..

[52] Archer S.L., Kulik T.J., Chesler E., Weir E.K. (1986). The effects of substance P on the preconstricted pulmonary vasculature of the anesthetized dog. Proc. Soc. Exp. Biol. Med..

[53] Regoli D., Drapeau G., Dorleans-Just P. (1987). Pharmacological receptors for substance P and neurokinins. Life Sci..

[54] Sung J., Ramnath D., Tamizhselvi R., Bhatia M. (2008). Neurokinin A engages neurokinin-1 receptor to induce NF-κB-dependent gene expression in murine macrophages: Implications of ERK1/2 and PI 3-kinase/Akt pathways. Am. J. Physiol. Cell Physiol..

[55] Hernandez J.M., Cox G., Janssen L.J. (2008). Involvement of the neurokinin-2 receptor in airway smooth muscle stretch-activated contractions assessed in perfused intact bovine bronchial segments. J. Pharmacol. Exp. Ther..

[56] Dion S., Rouissi N., Nantel F., Jukic D., Rhaleb N.E., Tousignant C., Telemaque S., Drapeau G., Regoli D., Naline E. (1990). Structure-activity study of neurokinins: Antagonists for the neurokinin-2 receptor. Pharmacology.

[57] Gallicchio M., Rosa A.C., Benetti E., Collino M., Dianzani C., Fantozzi R. (2006). Substance P-induced cyclooxygenase-2 expression in human umbilical vein endothelial cells. Br. J. Pharmacol..

[58] Haley K.J., Sunday M.E., Osathanondh R., Du J., Vathanaprida C., Karpitsky V.V., Krause J.E., Lilly C.M. (2001). Developmental expression of neurokinin A and functional neurokinin-2 receptors in lung. Am. J. Physiol. Lung Cell. Mol. Physiol..

[59] Couture R., Laneuville O., Guimond C., Drapeau G., Regoli D. (1989). Characterization of the peripheral action of neurokinins and neurokinin receptor selective agonists on the rat cardiovascular system. Naunyn-Schmiedeberg’s Arch. Pharmacol..

[60] Kotecha N., Neild T.O. (1995). Actions of vasodilator nerves on arteriolar smooth muscle and neurotransmitter release from sympathetic nerves in the guinea-pig small intestine. J. Physiol..

[61] Kawasaki H. (2002). Regulation of vascular function by perivascular calcitonin gene-related peptide-containing nerves. Jpn. J. Pharmacol..

[62] Kawasaki H., Nuki C., Saito A., Takasaki K. (1990). Adrenergic modulation of calcitonin gene-related peptide (CGRP)-containing nerve-mediated vasodilation in the rat mesenteric resistance vessel. Brain Res..

[63] Donoso M.V., Hermosilla D., Navarrete C., Alzvarez P., Lillo J.G., Huidobro-Toro J.P. (2012). Reciprocal sympatho-sensory control: Functional role of nucleotides and calcitonin gene-related peptide in a peripheral neuroeffector junction. Neuroscience.

[64] Leslie K.O. (2012). Idiopathic fibrosis may be a disease of recurrent, tractional injury to the periphery of the aging lung: A unifying hypothesis regarding etiology and pathogenesis. Arch. Pathol. Lab. Med..

[65] Ogawa T., Shichino S., Ueha S., Matsushima K. (2021). Macrophages in lung fibrosis. Int. Immunol..

[66] Verleden S.E., Tanabe N., McDonough J.E., Vasilescu D.M., Xu F., Wuyts W.A., Piloni D., De Sadeleer L., Willems S., Mai C. (2020). Small airways pathology in idiopathic pulmonary fibrosis: A retrospective cohort study. Lancet Respir. Med..

[67] Narula M., McGovern A.E., Yang S., Farrell M.J., Mazzone S.B. (2014). Afferent neural pathways mediating cough in animals and humans. J. Thorac. Dis..

[68] Ochoa-Callejero L., Garcia-Sanmarin J., Villoslada-Blanco P., Iniguez M., Perez-Matute P., Pujada E., Fowkes M.E., Brody R., Oteo J.A., Martinez A. (2021). Circulating levels of calcitonin gene-related peptide are lower in COVID-19 patients. J. Endoc Soc..

[69] Undem B.J., Nassenstein C. (2009). Airway nerves and dyspnea associated with inflammatory airway disease. Respir. Physiol. Neurobiol..

[70] Zhou Y., Zhang M., Sun G.Y., Liu Y.P., Ran W.Z., Peng L., Guan C.X. (2013). Calcitonin gene-related peptide promotes the wound healing of human bronchial epithelial cells via PKC and MAPK pathways. Regul. Pept..

[71] Kawanami Y., Morimoto Y., Kim H., Nakamura T., Machida K., Kido T., Asonuma E., Yatera K., Yoshii C., Kido M. (2009). Calcitonin gene-related peptide stimulates proliferation of alveolar epithelial cells. Respir. Res..

[72] Wei B., Sun M., Shang Y., Zhang C., Jiao X. (2018). Neurokinin 1 receptor promotes rat airway smooth muscle cell migration in asthmatic airway remodelling by enhancing tubulin expression. J. Thorac. Dis..

[73] Chang H.Y., Funayama H., Mizuta K., Emala C., Gallos G. (2013). Anti-proliferative effects of the neurokinin 1 receptor in human airway smooth muscle cells. Am. J. Respir. Crit. Care Med..

[74] Widdicombe J.G. (2003). Overview of neural pathways in allergy and asthma. Pulm. Pharmacol. Ther..

[75] Herrera J.A., Dingle L.A., Angeles Monetero M., Venkateswaran R.V., Blaikley J.F., Branato F., Pearson S., Lawless C., Thronton D.J. (2023). Morphologically intact airways in lung fibrosis have an abnormal proteome. Respir. Res..

[76] Cadieux A., Monast N.P., Pomerleau F., Fournier A., Lanoue C. (1999). Bronchoprotector properties of calcitonin gene-related peptide in guinea pig and human airways. Am. J. Respir. Crit. Care Med..

[77] Tanaka D.T., Grunstein M.M. (1984). Mechanisms of substance P-induced contraction of rabbit airway smooth muscle. J. Appl. Physiol. Respir. Environ. Exerc. Physiol..

[78] Guan M., Ying S., Wang Y. (2021). Increased expression of transient receptor potential channels and neurogenic factors associates with cough severity in a guinea pig model. BMC Pulm. Med..

[79] Ojiaku C.A., Yoo E.J., Panettieri R.A. (2017). Transforming growth factor β1 function in airway remodeling and hyperresponsiveness. The missing link?. Am. J. Respir. Cell Mol. Biol..

[80] Kendall R.T., Feghali-Bostwick C.A. (2014). Fibroblasts in fibrosis: Novel roles and mediators. Front. Pharmacol..

[81] Shochet G.E., Brook E., Barndenstein-Wald B., Shitrit D. (2020). TGF-β pathway activation by idiopathic pulmonary fibrosis (IPF) fibroblast derived soluble factors is mediated by IL-6 trans-signaling. Respir. Res..

[82] Jia M., Rosas L., Kapetanaki M.G., Tabib T., Sebrat J., Cruz T., Bondonese A., Mora A.L., Lafyatis R., Rojas M. (2023). Early events marking lung fibroblast transition to profibrotic state in idiopathic pulmonary fibrosis. Respir. Res..

[83] Yule K.A., White S.R. (1999). Migration of 3T3 and lung fibroblasts in response to calcitonin gene-related peptide and bombesin. Exp. Lung Res..

[84] Ramos C., Montano M., Cisneros J., Sommer B., Delgado J., Gonzalez-Avila G. (2007). Substance P up-regulates matrix metalloproteinase-1 and down-regulates collagen in human lung fibroblast. Exp. Lung Res..

[85] Gu Y., Lawrence T., Mohamed R., Liang Y., Yahaya B.H. (2022). The emerging roles of interstitial macrophages in pulmonary fibrosis: A perspective from scRNA-seq analyses. Front. Immunol..

[86] Duan J.X., Zhou Y., Zhou A.Y., Guan X.X., Liu T., Yang H.H., Xie H., Chen P. (2017). Calcitonin gene-related peptide exerts anti-inflammatory property through regulating murine macrophages polarization in vitro. Mol. Immunol..

[87] Zhu F., Yu D., Qin X., Qian Y., Ma J., Li W., Liu C., Wang C., Zhang Y., Li Y. (2023). The neuropeptide CGRP enters the macrophage cytosol to suppress the NLRP3 inflammasome during pulmonary infection. Cell. Mol. Immunol..

[88] Deng T., Yang L., Zheng Z., Li Y., Ren W., Wu C., Guo L. (2017). Calcitonin gene-related peptide induces IL-6 expression in RAW264.7 macrophages mediated by mmu_circRNA_007893. Mol. Med. Rep..

[89] Joos G.F., Germonpre P.R., Kips J.C., Peleman R.A., Pauwels R.A. (1994). Sensory neuropeptides and the human lower airways: Present state and future directions. Eur. Respir. J..

[90] Shen W., Wang X., Xiang H., Shichi S., Nakamoto H., Kimura S., Sugiyama K., Taketomi A., Kitamura H. (2023). IFN-γ–STAT1-mediated NK2R expression is involved in the induction of antitumor effector CD8+ T cells in vivo. Cancer Sci..

[91] Zhao X.N., Bai Z.Z., Li C.H., Sheng C.L., Li H.Y. (2020). The NK-1R antagonist Aprepitant prevents LPS-induced oxidative stress and inflammation in RAW264.7 macrophages. Drug Des. Dev. Ther..

[92] Network I.P.F.C.R., Raghu G., Anstrom K.J., King T.E., Lasky J.A., Martinez F.J. (2012). Prednisone, azathioprine, and N-acetylcysteine for pulmonary fibrosis. N. Engl. J. Med..

[93] Polverino M., Polverino F., Fasolino M., Ando F., Alfieri A., De Blasio F. (2012). Anatomy and neuro-pathophysiology of the cough reflex arc. Multidiscip. Respir. Med..

[94] Belvisi M.G., Birrell M.A. (2017). The emerging role of transient receptor potential channels in chronic lung disease. Eur. Respir. J..

[95] Young E.C., Smith J.A. (2011). Pharmacologic therapy for cough. Curr. Opin. Pharmacol..

[96] Ryerson C.J., Abbritti M., Ley B., Elicker B.M., Jones K.D., Collard H.R. (2011). Cough predicts prognosis in idiopathic pulmonary fibrosis. Respirology.

[97] Hope-Gill B.D.M., Hilldrup S., Davies C., Newton R.P., Harrison N.K. (2003). A study of the cough reflex in idiopathic pulmonary fibrosis. Am. J. Respir. Crit. Care Med..

[98] Harrison N.K. (2004). Idiopathic pulmonary fibrosis: A nervous cough?. Pulm. Pharmacol. Ther..

[99] Jones R.M., Hilldrup S., Hope-Gill B.D.M., Eccles R., Harrison N.K. (2011). Mechanical induction of cough in idiopathic pulmary fibrosis. Cough.

[100] Ricci A., Graziano P., Bronzetti E., Saltini C., Sciancchitano S., Cherubini E., Renzoni E., Du Bois R.M., Grutters J.C., Mariotta S. (2007). Increased pulmonary neurotrophin protein expression in idiopathic interstitial pheumonias. Sarcoidosis Vasc. Diffus. Lung Dis..

[101] Wakwaya Y., Ramdurai D., Swigris J.J. (2021). Managing cough in idiopathic pulmonary fibrosis. Chest.

[102] George P.M., Mitchell J.A. (2019). Defining a pathological role for the vasculature in the development of fibrosis and pulmonary hypertension in interstitial lung disease. Am. J. Physiol. Lung Cell. Mol. Physiol..

[103] Ryu J.H., Krowka M.J., Pellikka P.A., Swanson K.L., McGoon M.D. (2007). Pulmonary hypertension in patients with interstitial lung diseases. Mayo Clin. Proc..

[104] Strange C., Highland K.B. (2005). Pulmonary hypertension in interstitial lung disease. Curr. Opin. Pulm. Med..

[105] Lettieri C.J., Nathan S.D., Barnett S.D., Ahmed S., Shorr A.F. (2018). Prevalence and outcomes of pulmonary arterial hypertension in advanced idiopathic pulmonary fibrosis. Chest.

[106] Nadrous H.F., Pellikka P.A., Krowka M.J., Swanson K.L., Chaowalit N., Decker P.A., Ryu J.H. (2005). Pulmonary hypertension in patients with idiopathic pulmonary fibrosis. Chest.

[107] Luff S.E. (1996). Ultrastructure of sympathetic axons and their structural relationship with vascular smooth muscle. Anat. Embryol..

[108] Tuder R.M. (2016). Pulmonary vascular remodeling in pulmonary hypertension. Cell Tissue Res..

[109] Jia Z., Wang S., Yan H., Cao Y., Zhang X., Wang L., Zhang Z., Lin S., Wang X., Mao J. (2023). Pulmonary vascular remodeling in pulmonary hypertension. J. Pers. Med..

[110] Iwasawa T., Kato S., Ogura T., Kusakawa Y., Iso S., Baba T., Fukui K., Oba M.S. (2014). Low-normal lung volume correlates with pulmonary hypertension in fibrotic idiopathic interstitial pneumonia: Computer-aided 3D quantitative analysis of chest CT. AJR Am. J. Roentgenol..

[111] Shekerdemian L., Bohn D. (1999). Cardiovascular effects of mechanical ventilation. Arch. Dis. Child..

[112] Sakao S., Tanabe N., Tatsumi K. (2019). Hypoxic pulmonary vasoconstriction and the diffusing capacity in pulmonary hypertension secondary to idiopathic pulmonary fibrosis. J. Am. Heart Assoc..

[113] Turner-Warwick M. (1963). Precapillary systemic-pulmonary anastomoses. Thorax.

[114] Keane M.P., Arenberg D.A., Lynch J.P., Whyte R.I., Iannettoni M.D., Burdick M.D., Wilke C.A., Morris S.B., Glass M.C., DiGiovine B. (1997). The CXC chemokines, IL-8 and IP-10, regulate angiogenic activity in idiopathic pulmonary fibrosis. J. Immunol..

[115] Golden A., Bronk T.T. (1953). Diffuse interstitial fibrosis of lungs. Arch. Intern. Med..

[116] Coalson J.J. (1982). The ultrastructure of human fibrosing alveolitis. Virchows Arch..

[117] Gracey D.R., Divertie M.B., Brown A.L. (1968). Alveolar-capillary membrane in idiopathic interstitial pulmonary fibrosis. Am. Rev. Respir. Dis..

[118] Ebina M., Shimizukawa M., Shibata N., Kimura Y., Suzuki T., Endo M., Sasano H., Kondo T., Nukiwa T. (2004). Heterogeneous increase in CD34-positive alveolar capillaries in idiopathic pulmonary fibrosis. Am. J. Respir. Crit. Care Med..

[119] Renzoni E.A., Walsh D.A., Salmon M., Wells E.A., Sestini P., Nicholson A.G., Veeraraghyayan S., Bishop A.E., Romanska H.M., Pantelidids P. (2003). Interstitial vascularity in fibrosing alveolitis. Am. J. Respir. Crit. Care Med..

[120] Burdick M.D., Murray L.A., Keane M.P., Xue Y.Y., Zisman D.A., Belperio J.A., Strieter R.M. (2004). CXCL11 attenuates bleomycin-induced pulmonary fibrosis via inhibition of vascular remodeling. Am. J. Respir. Crit. Care Med..

[121] Stockman C., Kerdiles Y., Nomaksteinsky M., Weidemann A., Takeda N., Doedens A., Torres-Collado A.X., Iruela-Arispe L., Nizet V., Johnson R.S. (2010). Loss of myeloid cell-derived vascular endothelial growth factor accelerates fibrosis. Proc. Natl. Acad. Sci. USA.

[122] Oliveira A.C., Fu C., Lu Y., Williams M.A., Pi L., Brantly M.L., Ventetuolo C.E., Raizada M.K., Mehrad B., Scott E.W. (2019). Chemokine signaling axis between endothelial and myeloid cells regulates development of pulmonary hypertension associated with pulmonary fibrosis and hypoxia. Am. J. Physiol. Lung Cell. Mol. Physiol..

[123] Suga M., Iyonaga K., Okamoto T., Gushima Y., Miyakawa H., Akaike T., Ando M. (2000). Characteristic elevation of matrix metalloproteinase activity inidiopathic interstitial pneumonias. Am. J. Respir. Crit. Care Med..

[124] Champion H.C., Bivalacqua T.J., Toyoda K., Heistad D.D., Hyman A.L., Kadowitz P.J. (2000). In vivo gene transfer of prepro-calcitonin gene-related peptide to the lung attenuates chronic hypoxia-induced pulmonary hypertension in the mouse. Circulation.

[125] Zhao Q., Liu Z., Wang Z., Yang C., Lui J., Lu J. (2007). Effect of prepro-calcitonin gene related peptide-expressing endothelial progenitor cells on pulmonary hypertension. Ann. Thorac. Surg..

[126] Chen K.H., Lai Y.L., Chen M.J. (2012). Oxygen radicals and substance P in perinatal hypoxia-exaggerated, monocrotaline-induced pulmonary hypertension. Chin. J. Physiol..

[127] Chen L.W., Chen C.F., Lai Y.L. (1999). Chronic activation of neurokinin-1 receptor induces pulmonary hypertension in rats. Am. J. Physiol..

[128] Springer J., Fisher A. (2003). Substance P-induced pulmonary vascular remodelling in precision cut lung slices. Eur. Respir. J..

[129] Tjen-a-looi S., Ekman R., Lippton H., Cary J., Keith I. (1992). CGRP and somatostatin modulate chronic hypoxic pulmonary hypertension. Am. J. Physiol. Heart Circ. Physiol..

[130] Bartosik I., Eskilsson J., Ekman R., Akesson A., Scheja A. (2002). Correlation between plasma concentrations of calcitonin gene related peptide and pulmonary pressure in patients with systemic schlerosis. Ann. Rheum. Dis..

[131] Maggi C.A., Giachetti A., Rey R.D., Said S.I. (1995). Neuropeptides as regulators of airway function: Vasoactive intestinal peptide and the tachykinins. Physiol. Rev..

[132] Mannan M.M., Springall D.R., Denard C., Moradoghli-Haftvani A., Eddahibi S., Adnot S., Polak J.M. (1995). Decreased endothelium-dependent pulmonary vasodilator effect of calcitonin gene-related peptide in hypoxic rats contrasts with increased binding sites. Eur. Respir. J..

[133] Lo C.C.W., Moosavi S.M., Bubb K.J. (2018). The regulation of pulmonary vascular tone by neuropeptides and the implications for pulmonary hypertension. Front. Physiol..

[134] Norton C.E., Grunz-Borgmann E.A., Hart M.L., Jones B.W., Franklin C.L., Boerman E.M. (2021). Role of perivascular nerve and sensory neurotransmitter dysfunction in inflammatory bowel disease. Am. J. Physiol. Heart Circ. Physiol..

[135] Chaudhary K.R., Deng Y., Yang A., Cober N.D., Stewart D.J. (2021). Penetrance of Severe Pulmonary Arterial Hypertension in Response to Vascular Endothelial Growth Factor Receptor 2 Blockade in a Genetically Prone Rat Model Is Reduced by Female Sex. J. Am. Heart Assoc..

[136] Avona A., Burgos-Vera C., Burton M.D., Akopian A.N., Price T.J., Dussor G. (2019). Dural calcitonin gene-related peptide produces female-specific responses in rodent migraine models. J. Neurosci..

[137] Shammout B., Johnson J.R. (2019). Pericytes in Chronic Lung Disease. Adv. Exp. Med. Biol..

[138] Hung C.F., Wilson C.L., Schnapp L.M. (2019). Pericyte Biology in Different Organs.

[139] Zhang Z., Zhou H.N., He S.S., Xue M.Y., Li T., Liu L.M. (2020). Research advances in pericyte function and their roles in diseases. Chin. J. Traumatol..

[140] Garrison A.T., Bignold R.E., Wu X., Johnson J.R. (2023). Pericytes: The lung-forgotten cell type. Front. Physiol..

[141] Hamid Q. (2003). Gross pathology and histopathology of asthma. J. Allergy Clin. Immunol..

[142] Simonneau G., Montani D., Celermajer D.S., Denton C.P., Gatzoulis M.A., Krowka M.J., Williams P.G., Souza R. (2019). Haemodynamic definitions and updated clinical classification of pulmonary hypertension. Eur. Respir. J..

[143] Crnkovic S., Valzano F., Flieber E., Gindlhuber J., Thekkekara Puthenparampil H., Basil M., Morley M.P., Katzen J., Gschwandtner E., Klepetko W. (2022). Single-cell transcriptomics reveals skewed cellular communication and phenotypic shift in pulmonary artery remodeling. JCI Insight.

[144] Yuan K., Liu Y., Zhang Y., Nathan A., Tian W., Yu J., Sweatt A.J., Shamshou E.A., Condon D., Chakraborty A. (2020). Mural Cell SDF1 Signaling Is Associated with the Pathogenesis of Pulmonary Arterial Hypertension. Am. J. Respir. Cell Mol. Biol..

[145] Yamaguchi M., Hirai S., Tanaka Y., Sumi T., Tada M., Takahashi H., Watanabe A., Sakuma Y. (2020). Pericyte-myofibroblast transition in the human lung. Biochem. Biophys. Res. Commun..

[146] Savai R., Pullamsetti S., Kolbe J., Bieniek E., Fink L., Scheed A., Ritter C., Dahal B.K., Vater A., Klussmannn S. (2012). Immune/inflammatory cell involvement in the pathology of idiopathic pulmonary arterieal hypertension. Am. J. Respir. Crit. Care Med..

[147] Johnson J.R., Folestad E., Rowley J.E., Noll E.M., Walker S.A., Lloyd C.M., Rankin S.M., Pietras K., Eriksson U., Fuxe J. (2015). Pericytes contribute to airway remodeling in a mouse model of chronic allergic asthma. Am. J. Physiol. Lung Cell. Mol. Physiol..

[148] Bignold R.E., Shammout B., Rowley J.E., Repici M., Simms J., Johnson J.R. (2022). Chemokine CXCL12 drives pericyte accumulation and airway remodeling in allergic airway disease. Respir. Res..

[149] Butsabong T., Felippe M., Campagnolo P., Maringer K. (2021). The emerging role of perivascular cells (pericytes) in viral pathogenesis. J. Gen. Virol..

[150] Ricard N., Tu L., Le Hiress L., Huerta A., Phan C., Thuillet R., Sattler C., Fadel E., Seferian A., Montani D. (2014). Increased pericyte coverage mediated by endothelial-derived fibroblast growth factor-2 and interleukin-6 is a source of smooth muscle-like cells in pulmonary hypertension. Circulation.

[151] Bordenave J., Thuillet R., Tu L., Phan C., Cumont A., Marsol C., Huertas A., Savale L., Hibert M., Galzi J.L. (2020). Neutralization of CXCL12 attenuates established pulmonary hypertension in rats. Cardiovasc. Res..

[152] Hung C., Linn G., Chow Y.H., Kobayashi A., Mittelsteadt K., Altemeier W.A., Sgharib S.A., Schnapp L.M., Duffield J.S. (2013). Role of lung pericytes and resident fibroblasts in the pathogenesis of pulmonary fibrosis. Am. J. Respir. Crit. Care Med..

[153] Tannenberg P., Chang Y.T., Muhl L., Lavina B., Gladh H., Genove G., Betsholtz C., Folestad E., Tran-Lundmark K. (2018). Extracellular retention of PDGF-B directs vascular remodeling in mouse hypoxia-induced pulmonary hypertension. Am. J. Physiol. Lung Cell. Mol. Physiol..

[154] Chetty A., Nielsen H.C. (2021). Targeting airway smooth muscle hypertrophy in asthma: An approach whose time has come. J. Asthma Allergy.

[155] Hamilton N.B., Attwell D., Hall C.N. (2010). Pericyte-mediated regulation of capillary diameter: A component of neurovascular coupling in health and disease. Front. Neuroenergetics.

[156] Gong C.X., Zhang Q., Xiong X.Y., Yuan J.J., Yang G.Q., Huang J.C., Liu J., Duan C.M., Xu R., Qui Z.M. (2022). Pericytes Regulate Cerebral Perfusion through VEGFR1 in Ischemic Stroke. Cell. Mol. Neurobiol..

[157] Meng Y.M., Jiang X., Zhao X., Meng Q., Wu S., Chen Y., Kong X., Qiu X., Su L., Huang C. (2021). Hexokinase 2-driven glycolysis in pericytes activates their contractility leading to tumor blood vessel abnormalities. Nat. Commun..

[158] Friebe A., Englert N. (2022). NO-sensitive guanylyl cyclase in the lung. Br. J. Pharmacol..

[159] Aue A., Englert N., Harrer L., Schwiering F., Gaab A., Konig P., Adams R., Schmitko A., Friebe A. (2023). NO-sensitive guanylyl cyclase discriminates pericyte-derived interstitial from intra-alveolar myofibroblasts in murine pulmonary fibrosis. Respir. Res..

[160] Brown L.S., Foster C.G., Courtney J.M., King N.E., Howells D.W., Sutherland B.A. (2019). Pericytes and Neurovascular Function in the Healthy and Diseased Brain. Front. Cell. Neurosci..

[161] Shimizu F., Sano Y., Saito K., Abe M.A., Maeda T., Haruki H., Kanda T. (2012). Pericyte-derived glial cell line-derived neurotrophic factor increase the expression of claudin-5 in the blood-brain barrier and the blood-nerve barrier. Neurochem. Res..

[162] Hong H.S., Kim S., Jin Y., Son Y. (2020). Substance P enhances the therapeutic effect of MSCs by modulating their angiogenic potential. J. Cell. Mol. Med..

[163] Gao X., Bayraktutan U. (2023). Substance P reversibly compromises the integrity and function of blood-brain barrier. Peptides.

[164] Almaca J., Weitz J., Rodriguez-Diaz R., Pereira E., Caicedo A. (2018). The Pericyte of the Pancreatic Islet Regulates Capillary Diameter and Local Blood Flow. Cell Metab..

[165] Traber D.L., Lentz C.W., Traber L.D., Herndon D.N. (1992). Lymph and blood flow responses in central airways. Am. Rev. Respir. Dis..

[166] Lange M., Enkhbaatar P., Traber D.L., Cox R.A., Jacob S., Mathew B.P., Hamahata A., Traber L.D., Herndon D.N., Hawkins H.K. (2009). Role of calcitonin gene-related peptide (CGRP) in ovine burn and smoke inhalation injury. J. Appl. Physiol..

[167] Ghabriel M.N., Lu M.X., Leigh C., Cheung W.C., Allt G. (1999). Substance P-induced enhanced permeability of dura mater microvessels is accompanied by pronounced ultrastructural changes, but is not dependent on the density of endothelial cell anionic sites. Acta Neuropathol..

[168] Nguyen L.S., Villablanca A.C., Rutledge J.C. (1995). Substance P increases microvascular permeability via nitric oxide-mediated convective pathways. Am. J. Physiol..

[169] Kato K., Dieguez-Hurtado R., Park D.Y., Hong S.P., Kato-Azuma S., Adams S., Stehling M., Trappmann B., Wrana J.L., Koh G.Y. (2018). Pulmonary pericytes regulate lung morphogenesis. Nat. Commun..

[170] Keith I.M. (2000). The role of endogenous lung neuropeptides in regulation of the pulmonary circulation. Physiol. Rev..

[171] Hainis K.D., Sznajder J.I., Schraufnagel D.E. (1994). Lung lymphatics cast from the airspace. Am. J. Physiol..

[172] Peao M.N., Aguas A.P., de Sa C.M., Pereira A.S., Grande N.R. (1993). Scanning electron microscopy of the deep lymphatic network of the murine lung as viewed in corrosion casts. Lymphology.

[173] Marchetti C., Poggi P., Clement M.G., Aguggini G., Piacentini C., Icaro-Cornaglia A. (1994). Lymphatic capillaries of the pig lung: TEM and SEM observations. Anat. Rec..

[174] Leak L.V. (1980). Lymphatic removal of fluids and particles in the mammalian lung. Environ. Health Perspect..

[175] Solari E., Marcozzi C., Ottaviani C., Negrini D., Moriondo A. (2022). Draining the pleural space: Lymphatic vessels facing the most challenging task. Biology.

[176] Stump B., Cui Y., Kidambi P., Lamattina A.M., El-Chemaly S. (2017). Lymphatic changes in respiratory diseases: More than just remodeling of the lung?. Am. J. Respir. Cell Mol. Biol..

[177] Meinecke A.K., Nagy N., Lago G.D., Kirmse S., Klose R., Schrodter K., Zimmermann A., Helfrich I., Rundqvist H., Theegarten D. (2012). Aberrant mural cell recruitment to lymphatic vessels and impaired lymphatic drainage in a murine model of pulmonary fibrosis. Blood.

[178] El-Chemaly S., Malide D., Zudaire E., Ikeda Y., Wieinberg B.A., Pacheco-Rodriguez G., Rosas I.O., Aparicio M., Ren P., MacDonald S.D. (2009). Abnormal lymphangiogenesis in idiopathic pulmonary fibrosis with insights into cellular and molecular mechanisms. Proc. Natl. Acad. Sci. USA.

[179] Ebina M., Shibata N., Ohta H., Hisata S., Tamada T., Ono M., Okaya K., Kondo T., Nukiwa T. (2010). The disappearance of subpleural and interlobular lymphatics in idiopathic pulmonary fibrosis. Lymphat. Res. Biol..

[180] Karpanen T., Alitalo K. (2008). Molecular biology and pathology of lymphangiogenesis. Annu. Rev. Pathol..

[181] Baluk P., Naikawadi R.P., Kim S., Rodriguez F., Choi D., Hong Y.K., Wolters P.J., McDonald D.M. (2020). Lymphatic proliferation ameliorates pulmonary fibrosis after lung injury. Am. J. Pathol..

[182] Baluk P., Tammela T., Ator E., Lubynska N., Achen M.G., Hicklin D.J., Jeltxch M., Petraova T.V., Pytowski B., Stacker S.A. (2005). Pathogenesis of persistent lymphatic vessel hyperplasia in chronic airway inflammation. J. Clin. Investig..

[183] Reed H.O., Wang L., Sonett J., Chen M., Yang J., Li L., Aradi P., Jakus Z., D’Armiento J., Hancock W.W. (2019). Lymphatic impairment leads to pulmonary tertiary lymphoid organ formation and alveolar damage. J. Clin. Investig..

[184] Trivedi A., Reed H.O. (2023). The lymphatic vasculature in lung function and respiratory disease. Front. Med..

[185] Jannaway M., Iyer D., Mastrogiacomo D.M., Li K., Sung D.C., Yang Y., Kahn M.L., Scallan J.P. (2023). VEGFR3 is required for button junction formation in lymphatic vessels. Cell Rep..

[186] Alitalo K. (2011). The lymphatic vasculature in disease. Nat. Med..

[187] Oliver G., Kipnis J., Randolph G.J., Harvey N.L. (2020). The Lymphatic Vasculature in the 21st Century: Novel Functional Roles in Homeostasis and Disease. Cell.

[188] Scallan J.P., Zawieja S.D., Castorena-Gonzalez J.A., Davis M.J. (2016). Lymphatic pumping: Mechanics, mechanisms and malfunction. J. Physiol..

[189] Olszewski W.L. (2008). Contractility patterns of human leg lymphatics in various stages of obstructive lymphedema. Ann. N. Y Acad. Sci..

[190] Zawieja S.D., Castorena-Gonzalez J.A., Gui P., Li M., Bulley S.A., Jaggar J.H., Rock J.R., Davis M.J. (2019). Ano1 mediates pressure-sensitive contraction frequency changes in mouse lymphatic collecting vessels. J. Gen. Physiol..

[191] Zawieja S.D., Pea G.A., Broyhill S.E., Patro A., Bromert K.H., Li M., Norton C.E., Castorena-Gonzalez J.A., Hancock E.J., Bertram C.D. (2023). IP3R1 underlies diastolic ANO1 activation and pressure-dependent chronotropy in lymphatic collecting vessels. J. Gen. Physiol..

[192] Papp R., Nagaraj C., Zabini D., Nagy B.M., Lengyel M., Mauurer D.S., Sharma N., Egemnazarov B., Kovacs G., Kwapiszewska G. (2019). Targeting TMEM16A to reverse vasoconstriction and remodelling in idiopathic pulmonary arterial hypertension. Eur. Respir. J..

[193] Xie J., Liu W., Lv W., Han X., Kong Q., Wu Y., Liu X., Han Y., Shi C., Jia X. (2020). Transmembrane protein 16A/anoctamin 1 inhibitor T16Ainh-A01 reversed monocrotaline-induced rat pulmonary arterial hypertension. Pulm. Circ..

[194] Shang L., Wang K., Liu D., Qin S., Huang J., Zhao Y., Pang Y. (2020). TMEM16A regulates the cell cycle of pulmonary artery smooth muscle cells in high-flow-induced pulmonary arterial hypertension rat model. Exp. Ther. Med..

[195] McHale N.G., Thornbury K. (1986). A method for studying lymphatic pumping activity in conscious and anaesthetized sheep. J. Physiol..

[196] Datar S.A., Johnson E.G., Oishi P.E., Johengen M., Tang E., Aramburo A., Barton J., Kuo H.C., Bennet S., Xoinis K. (2012). Altered lymphatics in an ovine model of congenital heart disease with increased pulmonary blood flow. Am. J. Physiol. Lung Cell. Mol. Physiol..

[197] Scallan J.P., Knauer L.A., Hou H., Castorena-Gonzalez J.A., Davis M.J., Yang Y. (2021). Foxo1 deletion promotes the growth of new lymphatic valves. J. Clin. Investig..

[198] Castorena-Gonzalez J.A. (2022). Lymphatic valve dysfunction in western diet-fed mice: New insights into obesity-induced lymphedema. Front. Pharmacol..

[199] Bromley S.K., Thomas S.Y., Luster A.D. (2005). Chemokine receptor CCR7 guides T cell exit from peripheral tissues and entry into afferent lymphatics. Nat. Immunol..

[200] Marchal-Somme J., Uzunhan Y., Marchan-Adam S., Valeyre D., Soumelis V., Crestani B., Soler P. (2006). Cutting edge: Nonproliferating mature immune cells form a novel type of organized lymphoid structure in idiopathic pulmonary fibrosis. J. Immunol..

[201] Pierce E.M., Carpenter K., Jakubzick C., Kunkel S.L., Evanoff H., Flaherty K.R., Martinez F.J., Toews G.B., Hogaboam C.M. (2007). Idiopathic pulmonary fibrosis fibroblasts migrate and proliferate to CC chemokine ligand 21. Eur. Respir. J..

[202] Yamada K., Hoshino T. (1996). An examination of the close relationship between lymphatic vessels and nerve fibers containing calcitonin gene-related peptide and substance P in rat skin. Nagoya J. Med. Sci..

[203] Huang S., Ziegler C.G.K., Austin J., Mannoun N., Vukovic M., Ordovas-Montanes J., Shalek A.K., von Andrian U.H. (2021). Lymph nodes are innervated by a unique population of sensory neurons with immunomodulatory potential. Cell.

[204] Wang P.L., Czepielewski R.S., Randolph G.J. (2021). Sensory nerves regulate transcriptional dynamics of lymph node cells. Trends Immunol..

[205] Matsui S., Tanaka M., Kamiyoshi A., Sakurai T., Ichikawa-Shindo Y., Kawate H., Dai K., Cui N., Wei Y., Tanaka M. (2019). Endogenous calcitonin gene-related peptide deficiency exacerbates postoperative lymphedema by suppressing lymphatic capillary formation and M2 macrophage accumulation. Am. J. Pathol..

[206] An Y., Li Y., Chang W., Gao F., Ding X., Xu W., Han D. (2019). Quantitative Evaluation of the Function of the Sensory Nerve Fibers of the Palate in Patients with Obstructive Sleep Apnea. J. Clin. Sleep Med..

[207] Karuga F.F., Kaczmarski P., Szmyd B., Bialasiewicz P., Sochal M., Gabryelska A. (2022). The association between idiopathic pulmonary fibrosis and obstructive sleep apnea: A systematic review and meta-analysis. J. Clin. Med..

[208] Lancaster L.H., Mason W.R., Parnell J.A., Rice T.W., Loyd J.E., Milstone A.P., Collard H.R., Malow B.A. (2009). Obstructive Sleep Apnea Is Common in Idiopathic Pulmonary Fibrosis. Chest.

[209] Tudorache V., Traila D., Marc M., Oancea C., Manolescu D., Tudorache E., Timar B., Albai A., Fira-Mladinescu O. (2019). Impact of moderate to severe obstructive sleep apnea on the cognition in idiopathic pulmonary fibrosis. PLoS ONE.

[210] Mermigkis C., Bouloukaki I., Antoniou K., Papadogiannis G., Giannarakis I., Varouchakis G., Siafakas N., Schiza S.E. (2015). Obstructive sleep apnea should be treated in patients with idiopathic pulmonary fibrosis. Sleep Breath..

[211] Perlman C.E., Bhattacharya J. (2007). Alveolar expansion imaged by optical sectioning microscopy. J. Appl. Physiol..

[212] Ichimura H., Parthasarathi K., Quadri S., Issekutz A.C., Bhattacharya J. (2003). Mechano-oxidative coupling by mitochondria induces proinflammatory responses in lung venular capillaries. J. Clin. Investig..

[213] Kim T.H., Heo I.R., Kim C.K. (2022). Impact of high-risk of obstructive sleep apnea on chronic cough: Data from the Korea National Health and Nutrition Examination Survey. BMC Pulm. Med..

[214] Snow J.B., Norton C.E., Sands M.A., Weise Cross L., Yan S., Herbert L.M., Sheak J.R., Gonzales Bosc L.V., Walker B.R., Kanagy N.L. (2020). Intermittent hypoxia aurments pulmonary vasoconstrictor reactivity through PCKB/mitochondrial oxidant signaling. Am. J. Respir. Cell Mol. Biol..

[215] Snow J.B., Kitzis V., Norton C.E., Torres S.N., Johnson K.D., Kanagy N.L., Walker B.R., Resta T.C. (2008). Differential effects of chronic hypoxia and intermittent hypocapnic and eucapnic hypoxia on pulmonary vasoreactivity. J. Appl. Physiol..

[216] Glass D.S., Grossfield D., Renna H.A., Agarwala P., Spiegler P., DeLeon J., Reiss A.B. (2022). Idiopathic pulmonary fibrosis: Current and future treatment. Clin. Respir. J..

